# Association of plasma mitochondrial DNA with COPD severity and progression in the SPIROMICS cohort

**DOI:** 10.1186/s12931-021-01707-x

**Published:** 2021-04-26

**Authors:** William Z. Zhang, Katherine L. Hoffman, Kristen T. Schiffer, Clara Oromendia, Michelle C. Rice, Igor Barjaktarevic, Stephen P. Peters, Nirupama Putcha, Russell P. Bowler, J. Michael Wells, David J. Couper, Wassim W. Labaki, Jeffrey L. Curtis, Meilan K. Han, Robert Paine, Prescott G. Woodruff, Gerard J. Criner, Nadia N. Hansel, Ivan Diaz, Karla V. Ballman, Kiichi Nakahira, Mary E. Choi, Fernando J. Martinez, Augustine M. K. Choi, Suzanne M. Cloonan

**Affiliations:** 1 Division of Pulmonary and Critical Care Medicine, Joan and Sanford I. Weill Department of Medicine, Weill Cornell Medicine, New York, NY USA; 2 Weill Cornell Medicine, New York-Presbyterian Hospital, New York, NY USA; 3 Department of Population Health Science, Division of Biostatistics and Epidemiology, Weill Cornell Medicine, New York, NY USA; 4 Division of Nephrology and Hypertension, Joan and Sanford I. Weill Department of Medicine, Weill Cornell Medicine, New York, NY USA; 5 Division of Pulmonary and Critical Care Medicine, University of California Los Angeles Medical Center, Los Angeles, CA USA; 6 Pulmonary, Critical Care, Allergy, and Immunologic Medicine, Wake Forest School of Medicine, Winston-Salem, NC USA; 7 Johns Hopkins University School of Medicine, Baltimore, MD USA; 8 Division of Pulmonary, Critical Care and Sleep Medicine, National Jewish Health, Denver, CO USA; 9 University of Alabama at Birmingham, Birmingham, AL USA; 10 Department of Biostatistics, University of North Carolina at Chapel Hill, Chapel Hill, NC USA; 11 Division of Pulmonary and Critical Care Medicine, University of Michigan Health System, Ann Arbor, MI USA; 12 Section of Pulmonary and Critical Care Medicine, Salt Lake City Department of Veterans Affairs Medical Center, Salt Lake City, UT USA; 13 University of California at San Francisco, San Francisco, CA USA; 14 Department of Pulmonary & Critical Care Medicine, Temple University, Philadelphia, PA USA; 15 School of Medicine, Trinity Biomedical Sciences Institute, Trinity College Dublin, Ireland and Tallaght University Hospital, Dublin, Ireland; 16 Joan and Sanford I. Weill Department of Medicine, Weill Cornell Medicine, New York, USA

**Keywords:** COPD, Mitochondrial dysfunction, mtDNA, SPIROMICS

## Abstract

**Background:**

There is a lack of mechanism-driven, clinically relevant biomarkers in chronic obstructive pulmonary disease (COPD). Mitochondrial dysfunction, a proposed disease mechanism in COPD, is associated with the release of mitochondrial DNA (mtDNA), but plasma cell-free mtDNA has not been previously examined prospectively for associations with clinical COPD measures.

**Methods:**

P-mtDNA, defined as copy number of mitochondrially-encoded NADH dehydrogenase-1 (*MT-ND1*) gene, was measured by real-time quantitative PCR in 700 plasma samples from participants enrolled in the Subpopulations and Intermediate Outcome Measures in COPD Study (SPIROMICS) cohort. Associations between p-mtDNA and clinical disease parameters were examined, adjusting for age, sex, smoking status, and for informative loss to follow-up.

**Results:**

P-mtDNA levels were higher in participants with mild or moderate COPD, compared to smokers without airflow obstruction, and to participants with severe COPD. Baseline increased p-mtDNA levels were associated with better CAT scores in female smokers without airflow obstruction and female participants with mild or moderate COPD on 1-year follow-up, but worse 6MWD in females with severe COPD. Higher p-mtDNA levels were associated with better 6MWD in male participants with severe COPD. These associations were no longer significant after adjusting for informative loss to follow-up.

**Conclusion:**

In this study, p-mtDNA levels associated with baseline COPD status but not future changes in clinical COPD measures after accounting for informative loss to follow-up. To better characterize mitochondrial dysfunction as a potential COPD endotype, these results should be confirmed and validated in future studies.

*Trial Registration:* ClinicalTrials.gov NCT01969344 (SPIROMICS)

**Supplementary Information:**

The online version contains supplementary material available at 10.1186/s12931-021-01707-x.

## Introduction

Chronic obstructive pulmonary disease (COPD) is a chronic lung disease that is defined by airflow limitation on spirometry and persistent respiratory symptoms and is a leading cause of morbidity and mortality worldwide [1, 2]. This devastating impact of COPD stems from both an underdiagnosis in the community [3] and to an underlying heterogeneity of the disease, with the latter both in terms of clinical phenotypes as well as disease progression [4]. Given the relative lack of biomarkers that could predict important clinical events or discriminate between patients with different disease trajectories [5, 6], there has been a more recent call for endotype-driven research, to focus on pathobiological mechanisms for patient monitoring and treatment [7].

Among the relevant proposed mechanisms is mitochondrial dysfunction, with supporting evidence from both COPD patients [8–14] and experimental COPD models [15–18]. This dysfunction, partly attributed to mitochondrial oxidative stress [12] and manifested differently in different cell types, is often accompanied by the release of mitochondrial DNA (mtDNA) into the cytosol and extracellular space [19]. Intracellular mtDNA acts as a critical second messenger that activates the NOD-like receptor family, pyrin domain containing 3 (NLRP3) inflammasome and the cyclic GMP-AMP synthase (cGAS)-Stimulator of Interferon Genes (STING) system, which triggers a profound type I interferon innate immune response [20, 21]. Although less is known about extracellular mtDNA, circulating extracellular plasma mtDNA (p-mtDNA) has been associated with mortality in studies of intensive care unit populations, and there is some evidence to suggest that extracellular mtDNA release may be critical for the pathogenesis of some inflammatory diseases [22, 23]. MtDNA has been previously examined in a number of extracellular fluid compartments in COPD, including blood, urine, and exhaled breath condensate; however, these studies have shown both increased and decreased mtDNA levels [24, 25], leaving the role of this biomarker in COPD unclear [26–31]. Additionally, to our knowledge, p-mtDNA has not been evaluated for associations with other clinical measures of COPD severity or has been utilized to monitor disease progression in a large prospective COPD cohort.

Based on the hypothesis that extracellular mtDNA is reflective of mitochondrial dysfunction in subjects with COPD, we sought to determine if circulating p-mtDNA levels associate with clinical measures of COPD severity, using the Subpopulations and Intermediate Outcome Measures in COPD Study (SPIROMICS) cohort. Furthermore, given that SPIROMICS is an ongoing, longitudinal cohort, we examined whether baseline p-mtDNA levels can forecast disease progression, as represented by lung function and exercise tolerance decline and worsening of respiratory symptoms. Here we show that p-mtDNA levels are lower in individuals with severe COPD when compared to those with mild/moderate disease and are associated with subsequent changes in respiratory symptom burden and exercise in female participants. These findings may inform us about the trajectory of COPD from mild to severe disease, especially in females, and provides further supportive evidence for a role for mitochondrial dysfunction in COPD pathobiology.

## Methods

### Study design and sample collection

SPIROMICS (ClinicalTrials.gov NCT01969344) is an on-going longitudinal, prospective, multicenter observational study that recruited never smokers (≤ 1 pack-year of tobacco-smoking history), current or former smokers (ever-smokers, ≥ 20 pack-years) without airflow obstruction, and ever-smokers with airflow obstruction [32]. The individual institutional review boards (IRBs) of all participating clinical centers approved all study protocols, and all participants provided written informed consent. Clinical data were collected at the baseline and at follow-up study visits, including demographics, comorbidities, questionnaires, cigarette smoke exposure, spirometry, and 6-min walk distance (6MWD) [32]. Symptom burden was quantified with the COPD Assessment Test (CAT) and health-related quality of life (HRQL) with the St. George’s Respiratory Questionnaire (SGRQ) total score [33, 34]. The extent of emphysema was characterized using CT scans of the lung using Imbio diagnostics software, with percent emphysema defined as low attenuation area (LAA) less than − 950 Hounsfield Units at total lung capacity [35].

### Sample processing and quantification of P-mtDNA

Total DNA was isolated from plasma as described previously [20]. Briefly, 50 µL of plasma was diluted with 170 µL of PBS, and then centrifuged at 700×*g* at 4 °C for 5 min to remove cells and cellular debris. The obtained supernatant was further centrifuged at 18,000×*g* at 4 °C for 15 min, and the resulting supernatant (170 µl) was carefully saved. DNA was isolated from plasma using the DNeasy Blood and Tissue Kit (#69504; Qiagen). 200 µL of the provided elution buffer was used to collect the DNA.

Prior to quantification, the DNA solution was diluted 1:5 with nuclease-free deionized, distilled water. MtDNA levels were measured in triplicate by SYBR Green dye-based qPCR assay using a PRISM 7500 sequence detection system (Applied Biosystems), using the following primer sequences for human NADH dehydrogenase 1 gene: forward 5′-ATACCCATGGCCAACCTC-3′, reverse 5′-GGGCCTTTGCGTAGTTGTAT-3′ [20]. The thermal profile was as follows: 2 min at 50 °C, 10 min at 95 °C, 40 cycles for 15 s at 95 °C, and 1 min at 60 °C. For absolute quantitation of mtDNA, a standard curve was generated from DNA plasmid constructs in serial dilutions (ORIGENE #SC101172 and GenScript NM_173708).

### Statistical analysis

Clinical characteristics of SPIROMICS participants for whom p-mtDNA were measured were compared to those of all SPIROMICS participants summarized using medians and interquartile intervals or counts and percentages as appropriate. P-mtDNA was analyzed on the log2 scale to account for right-skewed distributions. Associations between p-mtDNA and baseline characteristics were first computed using unadjusted Pearson’s correlation coefficients for continuous baseline characteristics and ANOVA with Tukey’s post hoc adjustments for multiple comparisons for categorical baseline characteristics. We then examined whether p-mtDNA measured at baseline correlated with post-bronchodilator FEV_1_% predicted, 6MWD, SGRQ, CAT, and % emphysema measured at baseline using Pearson’s correlation coefficients and Pearson’s partial correlation coefficients holding age, sex, and current smoking status constant. Based on findings from our previous work which showed associations with urine mtDNA that differed among sex and COPD subgroups, we, where appropriate, stratified our p-mtDNA analyses by these same subgroups to determine if similar differential associations existed [30]. We next tested whether p-mtDNA measured at baseline correlated with changes between baseline and 1- and 3-year follow-up visit measurements of FEV_1_% predicted, 6MWD, SGRQ, and CAT, again using Pearson’s unadjusted and partial correlations, overall and within COPD and sex subgroups. These correlations with changes in COPD measures over time were further adjusted for informative loss to follow-up using Targeted Maximum Likelihood Estimation (TMLE) [36]. The mechanisms for loss to follow-up were explored by comparing baseline characteristics of patients who did and did not have 1- and 3-year follow up measurements using medians and interquartile intervals or counts and percentages as appropriate. The effect sizes of all estimates are reported as a correlation coefficient between -1 and 1 with a corresponding 95% confidence interval, representing the direction and strength of association between p-mtDNA and the clinical measures. Correlations reported as corrected for multiple comparisons were adjusted using the Benjamini and Hochberg False Discovery Rate (FDR) correction [37]. In a final analysis, we tested the association between baseline p-mtDNA and mortality using Cox Proportional Hazard models with robust standard errors, adjusting for age, sex, and smoking status. Analysis was conducted in R [38] and figures were produced using the package ggplot2 [39].

## Results

### Study population characteristics

**Fig. 1:**
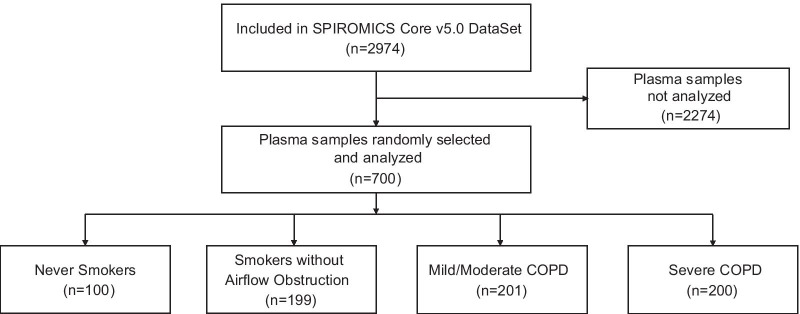
Flow diagram for the participants in the SPIROMICS Plasma mtDNA study. COPD, chronic obstructive pulmonary disease

A flow diagram for this study and the characteristics of the study participants for whom p-mtDNA levels were measured are shown in Fig. 1 and Table 1, respectively. Although samples were randomly selected, compared to the overall SPIROMICS population, the p-mtDNA study population was over-represented by non-smokers and participants with severe COPD, and under-represented in participants with mild or moderate COPD (p < 0.001, Additional file 1: Table 1); the study population was also less likely to be currently smoking compared to the overall SPIROMICS cohort (p = 0.002). Despite this difference, the sampled p-mtDNA population is otherwise representative of the overall SPIROMICS cohort in other clinical measures of COPD severity, including mean FEV_1_% predicted, 6MWD, SGRQ, CAT (Additional file 1: Table 1). As samples for p-mtDNA measurement were selected in 2018, these study participants have more complete long-term follow-up data compared to the overall SPIROMICS cohort (Additional file 1: Table 1).

**Table 1:** Baseline characteristics

Parameter	Non-smokers	Smokers without airflow obstruction	Mild/Mod COPD	Severe COPD	P-value*
	(n = 100)	(n = 199)	(n = 201)	(n = 200)	
Age [IQI]	56 [50, 65]	61 [52, 67]	66 [ 60, 71]	65 [59, 71]	< 0.001
Sex N (%)					
Male	42 (42%)	96 (48%)	130 (65%)	122 (61%)	< 0.001
Race N (%)					< 0.001
Asian	3 (3.1%)	3 (1.5%)	5 (2.5%)	2 (1.0%)	
Black	26 (27%)	52 (27%)	23 (12%)	29 (15%)	
Other	4 (4.1)	8 (4.1%)	4 (2.0%)	4 (2.0%)	
White	65 (66%)	133 (68%)	168 (84%)	163 (82%)	
Current smoking N (%)	0 (0%)	98 (48%)	77 (39%)	40 (20%)	< 0.001
FEV_1_% predicted [IQI]	102 [95, 109]	97 [86, 106]	71 [61, 80]	34 [27, 43]	< 0.001
6MWD [IQI]	465 [392, 516]	450 [390, 503]	429 [366, 484]	315 [249, 396]	< 0.001
CAT [IQI]	3 [1, 8]	9 [5, 16]	13 [8, 20]	19 [13, 24]	< 0.001
SGRQ [IQI]	5 [4, 11]	17 [8, 38]	28 [16, 45]	47 [39, 60]	< 0.001

*IQI* interquartile interval, *FEV*_*1*_ forced expiratory volume in 1 s, *6MWD* six-minute walk distance, *SGRQ* St. George’s Respiratory Questionnaire, *CAT* COPD Assessment Test

*Kruskal–Wallis test, Chi-square test, or Fisher’s exact test comparing participants within each subgroup, as appropriate

### P-mtDNA levels are lower in severe COPD compared to mild or moderate COPD

**Fig. 2:**
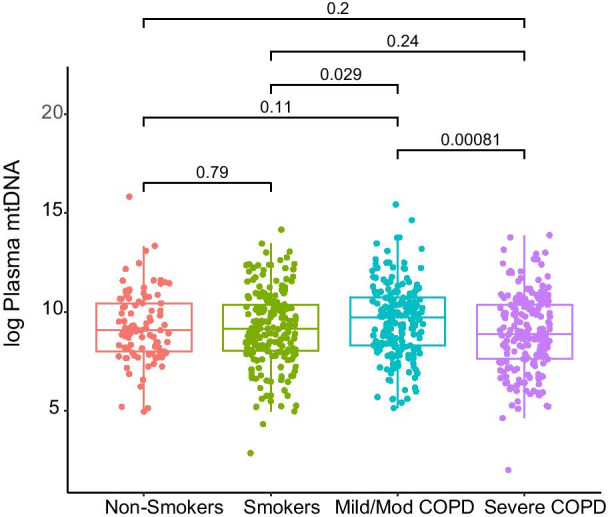
Plasma mtDNA levels were higher in subjects with mild/moderate COPD but falls in severe disease. P-mtDNA was measured in SPIROMICS participants, including never smokers (n = 100; red), smokers without airflow obstruction (n = 199; green), participants with mild/moderate COPD (n = 201; blue), and severe COPD (n = 200; purple). Data are presented as median with box indicating upper and lower quartiles, whiskers indicating extrema, and with p-values calculated by Tukey’s range test

We first examined the relationship between baseline p-mtDNA and characterization of COPD severity, with mild/moderate COPD defined as FEV_1_% predicted greater than 50%, and severe COPD defined as less than 50% [32]. There were no significant differences in p-mtDNA levels between non-smokers and ever-smokers without COPD nor between non-smokers and participants with COPD (mild or severe) (Fig. 2). In unadjusted analyses, participants with mild or moderate COPD had higher p-mtDNA levels compared to ever-smokers without COPD (p = 0.029, Fig. 2), an association that was no longer significant after adjustment for age, sex, and current smoking status (Additional file 1: Table 2). Notably, participants with severe COPD had lower p-mtDNA levels compared with those with mild or moderate COPD (p < 0.001, Fig. 2), a finding which remained significant after adjustment and correction for multiple comparisons testing (Additional file 1: Table 2). These relationships were seen in both males-only and females-only subgroup analyses; p-mtDNA levels did not differ significantly between males and females overall (Additional file 1: Fig. 1A, B). P-mtDNA levels did not differ by smoking status (Additional file 1: Fig. 2). Associations between p-mtDNA levels and clinical COPD measures, such as FEV_1_% predicted, 6MWD, CAT and SGRQ scores, and extent of radiographic emphysema were examined, but did not reveal any statistically significant relationships (Additional file 1: Table 3). P-mtDNA levels were not associated with urine mtDNA levels in matched study participants (Additional file 1: Fig. 3).

### Higher baseline p-mtDNA levels are associated with slower symptom burden progression in female smokers and female participants with COPD

SPIROMICS is an ongoing, prospective COPD cohort, and has extensive longitudinal follow-up data of its study participants. We utilized these follow-up time points to assess if baseline p-mtDNA levels associated with changes in clinical COPD measures between the baseline and one year after baseline visit (700/700 eligible for follow-up). Overall, we did not find any significant correlations between p-mtDNA with changes in clinical COPD measures at one year (Table 2). In subgroup analyses, we found that higher baseline p-mtDNA associated with improved (lower) CAT scores in females (r = − 0.15, CI [− 0.28, − 0.02], p = 0.02), especially in female smokers without airflow obstruction (r = − 0.28, CI [− 0.50, − 0.06], p = 0.01) and females with mild or moderate COPD (r = − 0.29, CI [− 0.54, − 0.03], p = 0.01); these associations remained significant after adjustment for age and smoking status (Table 2). However, higher baseline p-mtDNA was also associated with a decline in 6MWD in female participants with severe COPD (r = − 0.33, CI [− 0.61, − 0.05], p = 0.02) in unadjusted and adjusted analyses, a relationship that was not observed in female smokers or females with mild/moderate disease (Table 2). In males, higher baseline p-mtDNA did not associate with CAT but associated with better (longer) 6MWD in male participants with severe COPD (r = 0.27, CI [0.06, 0.48], p = 0.02), an association that remained significant after adjustment for age and smoking status (Table 2).

**Table 2:** P-mtDNA and disease progression at 1-year follow-up

	Unadjusted	Adjusted for age & smoking status
	$r$(CI)*	$r$(CI)*
FEV_1_% predicted		
All^a^	0 (− 0.08, 0.07)	− 0.01 (− 0.09, 0.06)
Males	− 0.01 (− 0.1, 0.09)	− 0.01 (− 0.12, 0.09)
Male smokers	− 0.08 (− 0.25, 0.1)	− 0.08 (− 0.27, 0.1)
Male mild/mod	− 0.03 (− 0.23, 0.17)	− 0.05 (− 0.26, 0.15)
Male severe	0.15 (− 0.07, 0.36)	0.14 (− 0.08, 0.35)
Females	0 (− 0.11, 0.11)	− 0.01 (− 0.12, 0.1)
Female smokers	0.04 (− 0.16, 0.23)	0.04 (− 0.16, 0.23)
Female mild/mod	− 0.09 (− 0.32, 0.15)	− 0.11 (− 0.35, 0.13)
Female severe	0.10 (− 0.16, 0.36)	0.08 (− 0.18, 0.34)
6MWD (meters)		
All^a^	− 0.02 (− 0.1, 0.06)	− 0.01 (− 0.09, 0.07)
Males	0.03 (− 0.07, 0.14)	0.04 (− 0.07, 0.15)
Male smokers	− 0.01 (− 0.19, 0.16)	− 0.01 (− 0.18, 0.16)
Male mild/mod	− 0.06 (− 0.28, 0.16)	− 0.05 (− 0.27, 0.16)
Male severe	*0.27 (0.06, 0.48)*^*b*^	*0.27 (0.05, 0.48)*
Females	− 0.09 (− 0.21, 0.03)	− 0.09 (− 0.21, 0.04)
Female smokers	0.03 (− 0.17, 0.22)	0.03 (− 0.17, 0.22)
Female mild/mod	0.08 (− 0.16, 0.32)	0.08 (− 0.16, 0.32)
Female severe	**− 0.33 (− 0.61, − 0.05)**^**c**^	**− 0.30 (− 0.59, − 0.02)**
SGRQ		
All^a^	− 0.02 (− 0.1, 0.07)	− 0.03 (− 0.12, 0.06)
Males	− 0.02 (− 0.13, 0.09)	− 0.04 (− 0.16, 0.08)
Male smokers	− 0.07 (− 0.27, 0.13)	− 0.1 (− 0.3, 0.1)
Male mild/mod	0 (− 0.2, 0.2)	0 (− 0.2, 0.2)
Male severe	− 0.14 (− 0.39, 0.1)	− 0.16 (− 0.41, 0.08)
Females	− 0.02 (− 0.14, 0.11)	− 0.01 (− 0.14, 0.11)
Female smokers	− 0.15 (− 0.38, 0.08)	− 0.15 (− 0.38, 0.08)
Female mild/mod	0.08 (− 0.2, 0.35)	0.10 (− 0.18, 0.37)
Female severe	− 0.02 (− 0.29, 0.25)	− 0.10 (− 0.39, 0.18)
CAT		
All^a^	− 0.07 (− 0.15, 0.01)	− 0.07 (− 0.15, 0.02)
Males	− 0.01 (− 0.11, 0.09)	− 0.01 (− 0.11, 0.1)
Male smokers	0.01 (− 0.16, 0.19)	0.04 (− 0.14, 0.21)
Male mild/mod	− 0.08 (− 0.29, 0.13)	− 0.08 (− 0.29, 0.12)
Male severe	− 0.05 (− 0.26, 0.17)	− 0.05 (− 0.27, 0.17)
Females	**− 0.15 (− 0.28, − 0.02)**	**− 0.14 (− 0.27, − 0.01)**
Female smokers	**− 0.28 (− 0.5, − 0.06)**	**− 0.30 (− 0.52, − 0.08)**
Female mild/mod	**− 0.29 (− 0.54, − 0.03)**	**− 0.27 (− 0.52, − 0.01)**
Female severe	0.05 (− 0.21, 0.31)	0.03 (− 0.22, 0.29)

*r* correlation coefficient, *CI* 95% confidence interval, *FEV*_*1*_ forced expiratory volume in 1 s, *6MWD* six-minute walk distance, *SGRQ* St. George’s Respiratory Questionnaire, *CAT* COPD Assessment Test

*Pearson’s correlation coefficients with log2 p-mtDNA as among all participants and within each group

^a^Adjusting for age, sex, and smoking status

^b^Italic—statistically significant with positive correlation coefficient

^c^Bold—statistically significant with negative correlation coefficient

At 3-year follow-up (566/700 eligible for follow-up), there was significant loss of study participants, and many of the associations observed at 1-year were no longer present. There were no statistically significant associations between p-mtDNA and the examined COPD outcomes, overall or in subgroups stratified by sex (Table 3). Additionally, we examined for associations between p-mtDNA and COPD exacerbation risk and did not find any significant relationships for either moderate (defined as exacerbations requiring treatment with systemic corticosteroids, antibiotics, or both) or severe (defined as exacerbations requiring emergency room visit or hospitalization) COPD exacerbations (Additional file 1: Fig. 4A, B). P-mtDNA levels did not differ in study participants who died during this follow-up period compared to those who did not, in unadjusted analysis or in the Cox proportional hazards model, adjusting for age, sex, and COPD status (Additional file 1: Fig. 5, Table 4).

**Table 3:** P-mtDNA and disease progression at 3-year follow-up

	Unadjusted	Adjusted for age & smoking status
	$r$(CI)*	$r$(CI)*
FEV_1_% predicted		
All^a^	0.04 (− 0.05, 0.14)	0.04 (− 0.06, 0.14)
Males	0.08 (− 0.05, 0.22)	0.09 (− 0.05, 0.23)
Females	0 (− 0.14, 0.13)	− 0.01 (− 0.15, 0.13)
6MWD (meters)		
All	− 0.04 (− 0.14, 0.07)	− 0.02 (− 0.12, 0.08)
Males	0 (− 0.13, 0.13)	0.03 (− 0.11, 0.17)
Females	− 0.08 (− 0.23, 0.07)	− 0.07 (− 0.23, 0.08)
SGRQ		
All	− 0.05 (− 0.16, 0.06)	− 0.06 (− 0.18, 0.05)
Males	− 0.05 (− 0.19, 0.10)	− 0.07 (− 0.22, 0.08)
Females	− 0.06 (− 0.22, 0.10)	− 0.06 (− 0.23, 0.10)
CAT		
All	0.01 (− 0.09, 0.11)	0 (− 0.09, 0.10)
Males	0.01 (− 0.12, 0.13)	− 0.01 (− 0.15, 0.12)
Females	0.02 (− 0.13, 0.16)	0.02 (− 0.12, 0.17)

^a^Adjusting for age, sex, and smoking status

*r* correlation coefficient, *CI* 95% confidence interval, *FEV*_*1*_ forced expiratory volume in 1 s, *6MWD* six-minute walk distance, *SGRQ* St. George’s Respiratory Questionnaire, *CAT* COPD Assessment Test

*Pearson’s correlation coefficients with log2 p-mtDNA as among all participants and within each group

### P-mtDNA is not associated with future changes in clinical COPD measures after adjusting for informative loss to follow-up

**Fig. 3:**
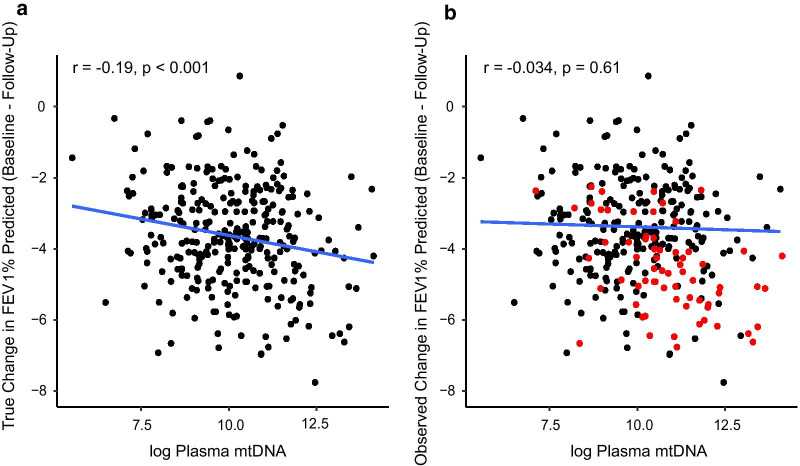
Illustrative example of informative missingness. A simulated correlation between p-mtDNA levels and FEV_1_% predicted in the true population (**a**) in the hypothetical, ideal situation where all participants had complete follow-up (**b**) in the hypothetical, more likely situation where patients with worse FEV_1_% were also less likely to have complete follow-up. Black dots represent complete cases, red dots indicate cases lost to follow-up

All longitudinal prospective cohort studies are prone to subject drop-out and non-response, and while the reason for dropping out can be either practical or biological, if missing data occur in a selective, non-random manner, the “missingness” can be informative and significantly impact data analysis and interpretation. When comparing the characteristics of participants with complete follow-up data with those missing data, we discovered that participants with missing follow-up FEV_1_% predicted at 1-year follow-up have more severe disease, by baseline FEV_1_% predicted (79% vs. 45%, p < 0.001, Additional file 1: Table 5) or by a number of other clinical measures, including 6MWD, CAT, and SGRQ (p < 0.001 in all comparisons, Additional file 1: Table 5). Similar differences were observed comparing participants with complete follow-up with those with missing FEV_1_% predicted at 3-year follow-up (Additional file 1: Table 6). This was consistently seen if we evaluated missingness by other variables, such as absent 6MWD, CAT, or SGRQ values at 1 or 3 years (not shown). A fraction of the loss to follow-up can be attributed to deaths, but death as an outcome represents only a minority of the those with missing follow-up data (Additional file 1: Tables 5–6). When we adjusted our analyses to account for missingness, the statistically significant associations observed at 1-year follow-up were no longer statistically significant (Additional file 1: Tables 7, 8). A simulated example showing the true data and the data observed (a subset dependent on severity of COPD) was generated for illustration (Fig. 3). The relevance of adjusting for missing data is apparent: if p-mtDNA is associated with COPD severity, and participants with more severe disease are more likely to be lost to follow-up, then analyzing only complete cases without adjusting for loss to follow-up would result in a biased conclusion.

## Discussion

In this large study of 700 participants from the SPIROMICS cohort, we tested the hypothesis that p-mtDNA, a surrogate for mitochondrial dysfunction, would associate with COPD severity and progression. To the best of our knowledge, this is the largest COPD population for which p-mtDNA has been evaluated. Here, we found that p-mtDNA levels were higher in participants with mild or moderate COPD compared to smokers without airflow obstruction, and to those with severe COPD. These findings are interesting and potentially supportive of a role for mitochondrial dysfunction in the transition from mild to severe disease in COPD.

The observation that p-mtDNA levels were lower in those with severe COPD when compared to individuals with mild/moderate disease has yet to be demonstrated in previous studies but is consistent with findings which show decreased mtDNA levels in the tissues of patients with severe COPD [24, 25]. P-mtDNA levels also trended higher in mild/moderate participants when compared to healthy smokers, suggesting a possible biphasic release of mtDNA that peaks in individuals with early disease but declines upon progression to severe disease. MtDNA is well recognized as a danger-associated molecular pattern (DAMP) that is an important component of the innate immune response [40], and is released by lung epithelial cells in response to cigarette smoke extract, likely as a result of mitochondrial stress [28, 29]. In vivo, short term smoke exposure increases bronchoalveolar lavage fluid mtDNA levels, and the number of abnormal mitochondria inside the lung epithelium increases with the length of smoke exposure [15, 28, 29]. The effect of prolonged cigarette smoke exposure on mtDNA levels has not been experimentally modeled, but perhaps with continued mitochondrial stress, and consequently, mtDNA release, mitochondrial exhaustion may occur. This phenomenon has been suggested in a number of diseases of dysregulated immunity, such as sepsis [41], as well as in diseases of accelerated aging, of which COPD is considered to be a prime example [42, 43]. Despite the lack of differences in p-mtDNA levels between healthy controls and individuals with mild/moderate or severe COPD, our findings may inform us as to the dynamic nature of p-mtDNA release in COPD disease trajectory whereby active p-mtDNA release plays a role in the course of the disease from healthy smokers to individuals with mild COPD, followed by a decline in p-mtDNA release that can be attributed to mitochondrial exhaustion in severe COPD.

Despite the above observations, we otherwise found no significant associations between p-mtDNA levels and baseline clinical measures of COPD and only minor associations between p-mtDNA and clinical correlates (CAT, 6MWD) in subgroup analyses at the one-year follow-up visit. We have previously demonstrated in a subset of this same cohort that higher urine mtDNA was associated with respiratory symptom burden and worse exercise tolerance, particularly in smokers without COPD and in women [30]. In this study, some of the statistically significant associations between p-mtDNA and 6MWD were similar to what we observed in our study of urine mtDNA, such as the association between higher baseline p-mtDNA levels and a lower 6MWD in women at 1 year [30]. Clinically, women with COPD report more dyspnea than men, and develop earlier COPD onset with more rapid lung function loss despite overall lower smoke exposure burden [44, 45]. In light of these observations, it is perhaps not coincidental that mtDNA is maternally inherited, which imparts a genetic risk and potentially provides some biological rationale for the sex-dependent associations we have found between mtDNA, CAT and 6MWD in this study.

One major strength of this current study is the assessment between p-mtDNA and longitudinal changes in COPD taking into consideration informative missingness in our analysis. This is important because significant changes in longitudinal measures may be missed or incorrect if only study participants with complete follow-up data are examined. We verified in our cohort that severity of illness was related to the likelihood of observing follow-up measures in our p-mtDNA cohort, as participants with worse baseline measures were significantly less likely to have subsequent 1- or 3-year COPD measurements recorded. We accounted for this in our correlations with TMLE by modeling the probability of observing the components of our longitudinal correlations (p-mtDNA measurements and the difference in COPD outcomes from baseline to follow-up) given the other baseline measures of severity of illness, before computing the correlation coefficient. Although some of our findings were no longer statistically significant after this process, our statistical adjustment for an informative missingness mechanism ensures that we are not overlooking important correlations or drawing biased conclusions in the relationship between p-mtDNA and COPD progression. In addition to modeling informative missingness, we chose our analytical method carefully, opting to examine differences between COPD measures at baseline and a single subsequent time point rather than a pooled model with more time points. Our modeling strategy was in response to our initial questions of interest, of whether p-mtDNA might associate with a change in COPD symptoms over two potential time frames: one relatively immediate (1-year follow-up) and one longer (3-year follow-up). Although there were data available to potentially examine the relationship between baseline p-mtDNA and COPD progression trajectories in more detail, given the several near-zero correlations in our current progression analyses, it is unlikely that a model pooling time points would add relevant new information.

There are still many other important unknowns regarding mtDNA and COPD, including the source of extracellular p-mtDNA. Although cigarette smoke is introduced in the airway, the multimorbidity of COPD suggests that the effect of cigarette smoke is systemic, and thus mtDNA could be released as a result of mitochondrial stress in epithelial cells [13, 29], circulating immune cells [8, 10], or endothelial cells [46] in the lung or elsewhere, or possibly a combination of these sources; the fact that mtDNA was detected in the urine of smokers and COPD patients is provocative supportive evidence for an extrapulmonary source of mtDNA [30]. Notably, urine mtDNA and p-mtDNA available on the same patients did not linearly associate in this study, indicating that the presence of mtDNA in urine is not simply a result of circulating p-mtDNA filtered by the kidney. We also did not find similar sex differences in p-mtDNA levels which we observed with females exhibiting higher urine mtDNA across the subgroups in the SPIROMICS cohort [30]. Thus, p-mtDNA and urine mtDNA may come from entirely different sources and represent distinct biological phenomena driving the results of these two studies.

Our study has several limitations. The smoking status was determined by self-report at baseline, corresponding to the baseline blood sample from which p-mtDNA levels were measured, and it is possible that a study participant could have stopped smoking at a future study visit, when additional clinical measurements were taken. In addition, we did not examine clinical parameters that may be more reflective of cachexia or sarcopenia (e.g. grip strength), phenotypes which are commonly observed in patients with severe COPD and which may be attributable to mitochondrial dysfunction [47, 48]. We performed many statistical tests in this study and recognize the potential for type I errors; we adjusted for multiple comparisons and the great majority of associations we observed were no longer statistically significant. We are also limited by the lack of a validation cohort, despite measuring p-mtDNA in a large number of SPIROMICS study participants; our findings should be examined and confirmed in future studies and in other COPD cohorts.

Over forty years after the seminal work of Fletcher and Peto, it is clear now that beyond accelerated lung function loss in smokers, there are multiple lung function trajectories that give rise to COPD, and that the natural history of COPD is not uniform but individualized in different patients [49, 50]. One of the aims of COPD research should be the development of tools and biomarkers that reflect the underlying pathobiology but could also track individualized disease progression; this is particularly important early at disease onset [7, 51]. While some studies have examined biomarkers in association with COPD exacerbations [5], fewer have evaluated biomarkers in relation to disease progression, given the difficulty in maintaining a large longitudinal cohort [52]. This study evaluated p-mtDNA as a conceptual marker of COPD progression, and observed possible interesting differences between p-mtDNA levels in individuals with mild COPD when compared to those with severe COPD. In subgroup analyses stratifying by sex, a number of associations between p-mtDNA and symptom burden as measured by 6MWD and CAT were observed, particularly in females; however, these observations must be considered as preliminary and require further testing. Nevertheless, the findings here add to the ever-expanding literature that suggests a role for mitochondrial dysfunction in severe COPD and advocates for future investigations measuring p-mtDNA in longitudinal studies.

## Supplementary Information

**Additional file 1: Figure 1.** P-mtDNA levels do not differ between males and females. **Figure 2.** P-mtDNA is not associated with smoking status. **Figure 3.** P-mtDNA is not associated with u-mtDNA. **Figure 4.** Baseline p-mtDNA levels do not differ between study participants with and without exacerbations. **Figure 5.** Baseline p-mtDNA levels do not differ by study participant mortality. **Table 1.** P-mtDNA cohort compared to overall SPIROMICS cohort. **Table 2.** P-mtDNA between subgroups. **Table 3.** P-mtDNA and clinical COPD measures. **Table 4.** P-mtDNA and all-cause mortality. **Table 5.** Baseline characteristics of participant with and without 1-year follow-up Spirometry Data. **Table 6.** Baseline characteristics of participant with and without 3-year follow-up Spirometry Data. **Table 7.** P-mtDNA and disease progression at 1-year follow-up, adjusted for missingness. **Table 8.** P-mtDNA and disease progression at 3-year follow-up, adjusted for missingness.

## Data Availability

Interested investigators may request access to available clinical data through processes outlined on the SPIROMICS website (https://www.spiromics.org/spiromics); additional data are available from the corresponding author on reasonable request.

## Abbreviations

6MWD: Six-minute walk distance
CAT: COPD assessment test
COPD: Chronic obstructive pulmonary disease
FEV_1_: Forced expiratory volume in one second
p-mtDNA: Plasma mitochondrial DNA
SGRQ: St. George’s Respiratory Questionnaire
TMLE: Targeted maximum likelihood estimation
