# Supplementary material for: Association of plasma mitochondrial DNA with COPD severity and progression in the SPIROMICS cohort
Source: Respir Res. 2021 Apr 26;22:126. doi: 10.1186/s12931-021-01707-x (PMC8074408; doi:10.1186/s12931-021-01707-x)
Supplement: Supplementary file 1 — Additional file 1: Figure 1. P-mtDNA levels do not differ between males and females. Figure 2. P-mtDNA is not associated with smoking status. Figure 3. P-mtDNA is not associated with u-mtDNA. Figure 4. Baseline p-mtDNA levels do not differ between study participants with and without exacerbations. Figure 5. Baseline p-mtDNA levels do not differ by study participant mortality. Table 1. P-mtDNA cohort compared to overall SPIROMICS cohort. Table 2. P-mtDNA between subgroups. Table 3. P-mtDNA and clinical COPD measures. Table 4. P-mtDNA and all-cause mortality. Table 5. Baseline characteristics of participant with and without 1-year follow-up Spirometry Data. Table 6. Baseline characteristics of participant with and without 3-year follow-up Spirometry Data. Table 7. P-mtDNA and disease progression at 1-year follow-up, adjusted for missingness. Table 8. P-mtDNA and disease progression at 3-year follow-up, adjusted for missingness. [file 12931_2021_1707_MOESM1_ESM.pdf]

SUPPLEMENTARY FIGURE LEGENDS

Supplementary Figure 1. P-mtDNA levels do not differ between males and females. P-mtDNA levels in COPD subgroups (A, male non-smokers n=42, male smokers n=96, male mild/moderate COPD n=130, male severe COPD n=122; female non-smokers n=58, female smokers n=103, female mild/moderate COPD n=71, female severe COPD n=78) and in males and females overall (B). Data are presented as median with box indicating upper and lower quartiles, whiskers indicating extrema, and with p-values calculated by Tukey's range test.

Supplementary Figure 2. P-mtDNA is not associated with smoking status. P-mtDNA levels in non-smokers (n=100), former smokers (n=377), and current smokers (n=211); of the 700 participants in whom p-mtDNA levels were measured, 12 had unknown smoking status. Data are presented as median with box indicating upper and lower quartiles, whiskers indicating extrema, and with p-values calculated by Tukey's range test.

Supplementary Figure 3. P-mtDNA is not associated with u-mtDNA. Log p-mtDNA and association with log u-mtDNA, tested with unadjusted linear regression models (never smokers n=47, smokers without airflow obstruction n=78, mild/moderate COPD n=93, severe COPD n=166). Unadjusted association was tested using Pearson's correlation coefficient.

Supplementary Figure 4. Baseline p-mtDNA levels do not differ between study participants with and without exacerbations. P-mtDNA levels stratified by number of moderate (A) and severe (B) exacerbations, in subgroups of 0 (moderate=391,

severe=521), 1-2 (moderate=154, severe=120), 3-5 (moderate=80, severe=40), and 6+ (moderate=64, severe=7). Data are presented as median with box indicating upper and lower quartiles, whiskers indicating extrema, and with p-values calculated by Spearman’s rank correlation test.

Supplementary Figure 5. Baseline p-mtDNA levels do not differ by study participant mortality. P-mtDNA levels in study participants who died during follow-up (n=48) and those who did not (n=642). Data are presented as median with box indicating upper and lower quartiles, whiskers indicating extrema, and with p-values calculated by Wilcoxon signed-rank test.

Supplementary Table 1. P-mtDNA Cohort Compared to Overall SPIROMICS Cohort.

Parameter	Plasma mtDNA Cohort (n = 700)	SPIROMICS Cohort (n = 2973)	P-value*
Age [IQI]	64 [56, 70]	64 [56 - 70]	0.2
Sex N (%)			0.2
Male	390 (56%)	1577 (53%)	
Female	310 (44%)	1397 47%)	
Race N (%)			0.5
Asian	5 (0.7%)	14 (0.5%)	
Black	130 (19%)	576 (19%)	
White	529 (76%)	2262 (77%)	
Baseline Current Smoking N (%)	211 (31%)	1092 (37%)	0.002
Subgroup N (%)			<0.001
Non-Smokers	100 (14%)	202 (6.8%)	
Smokers	199 (28%)	941 (32%)	
Mild/Moderate COPD	201 (29%)	1205 (41%)	
Severe COPD	200 (29%)	625 (21%)	
Baseline FEV_1 % Predicted [IQI]	77 [46, 96)	79 [55, 95]	0.09
Baseline 6MWD [IQI]	420 [345, 485]	411 [339, 475]	0.14
Baseline CAT [IQI]	12 [6, 20]	13 [7, 20]	0.4
Baseline SGRQ [IQI]	30 [12, 48]	30 [13, 48]	0.7
Eligible for 1-Year Follow-Up (%)	700 (100%)	2972 (100%)	>0.9
Completed 1-Year Follow-Up (%)	609 (87%)	2324 (78%)	<0.001
Eligible for 3-Year Follow-Up (%)	566 (81%)	1757 (59%)	<0.001
Completed 3-Year Follow-Up	384 (68%)	893 (51%)	<0.001

*Kruskal-Wallis test, Chi-square test, or Fisher's exact test comparing participants within each subgroup, as appropriate
Abbreviations: IQI=interquartile interval, FEV_1=forced expiratory volume in 1 second, 6MWD=six-minute walk distance, SGRQ=St. George's Respiratory Questionnaire, CAT=COPD Assessment Test

Supplementary Table 2. P-mtDNA between Subgroups.

Subgroups compared	Adjusted for age, sex & smoking status		Adjusted p-value for multiple comparisons
	$\hat{\beta}$ (CI)	*P-value**	*P-value**
Never smokers – Smokers without airflow obstruction	-0.07 (-0.62, 0.49)	0.81	0.81
Smokers without airflow obstruction – Mild/moderate COPD	0.34 (-0.05, 0.74)	0.085	0.26
Smokers without airflow obstruction – Severe COPD	-0.19 (-0.60, 0.21)	0.35	0.53
Mild/moderate COPD – Severe COPD	-0.55 (-0.93, -0.17)	0.005	0.029

*Pairwise contrasts of strata using Tukey's range test (unadjusted and adjusted)
Abbreviations: $\hat{\beta}$=estimated change log2 UACR with a one unit increase in clinical characteristic, CI=confidence interval

Supplementary Table 3. P-mtDNA and Clinical COPD Measures.

	Unadjusted r (CI)*	Adjusted for age & smoking status r (CI)*
FEV_1 % predicted	0.02 (-0.05, 0.09)	0.03 (-0.04, 0.11)
6MWD (meters)	0 (-0.07, 0.08)	0.02 (-0.06, 0.09)
SGRQ	-0.02 (-0.10, 0.05)	-0.02 (-0.10, 0.05)
CAT	-0.01 (-0.09, 0.06)	0.01 (-0.08, 0.06)
% Emphysema	-0.02 (-0.09, 0.05)	-0.03 (-0.10, 0.04)

*Pearson's correlation coefficients with log2 p-mtDNA as among all participants and within each group

Abbreviations: r=correlation coefficient, CI=95% confidence interval, FEV_1=forced expiratory volume in 1 second, 6MWD=six-minute walk distance, SGRQ=St. George's Respiratory Questionnaire, CAT=COPD Assessment Test

Supplementary Table 4. P-mtDNA and All-Cause Mortality.

Model	Hazard Ratio (CI)	P-value
Unadjusted	0.969 (0.831, 1.13)	0.686
Adjusted*	0.951 (0.813, 1.11)	0.531
Adjusted**	0.989 (0.851, 1.15)	0.888

Hazard ratios for all-cause mortality log2 p-mtDNA using Cox Proportional Hazard models with robust standard errors. *Adjusting for age, sex, and smoking status. **Adjusting for age, sex, smoking status, and subgroup.

Supplementary Table 5. Baseline Characteristics of Participant with and without 1-Year Follow-Up Spirometry Data.

Parameter	Complete Data (n = 609)	Missing V2 (n = 91)	P-value*
Age [IQI]	65 [56, 70]	62 [54, 68]	0.09
Sex N (%)			0.7
Male	337 (55%)	53 (58%)	
Female	272 (45%)	38 (42%)	
Race N (%)			0.2
Asian	10 (1.7%)	3 (3.4%)	
Black	108 (18%)	22 (25%)	
White	467 (77%)	62 (70%)	
Baseline Current Smoking N (%)	172 (29%)	39 (44%)	0.005
Baseline FEV_1 % Predicted [IQI]	79 [52, 97]	45 [31, 84]	<0.001
Baseline 6MWD [IQI]	427 [360, 490]	347 [261, 415]	<0.001
Baseline CAT [IQI]	12 [5, 19]	17 [10, 23]	<0.001
Baseline SGRQ [IQI]	27 [10, 45]	47 [28, 57]	<0.001
Deaths N (%)	0 (0%)	7 (7.7%)	< 0.001

*Kruskal-Wallis test, Chi-square test, or Fisher's exact test comparing participants within each subgroup, as appropriate
Abbreviations: IQI=interquartile interval, FEV_1=forced expiratory volume in 1 second, 6MWD=six-minute walk distance, SGRQ=St. George's Respiratory Questionnaire, CAT=COPD Assessment Test

Supplementary Table 6. Baseline Characteristics of Participant with and without 3-Year Follow-Up Spirometry Data.

Parameter	Complete Data (n = 384)	Missing V4 (n = 316)	P-value*
Age [IQI]	64 [56, 70]	65 [55, 70]	0.6
Sex N (%)			0.4
Male	209 (55%)	53 (57%)	
Female	176 (46%)	134 (43%)	
Race N (%)			>0.9
Asian	8 (2.1%)	5 (1.6%)	
Black	72 (19%)	58 (19%)	
White	289 (76%)	240 (77%)	
Baseline Current Smoking N (%)	108 (28%)	103 (34%)	0.2
Baseline FEV_1 % Predicted [IQI]	81 [58, 99]	66 [39, 92]	<0.001
Baseline 6MWD [IQI]	431 [368, 494]	396 [311, 464]	<0.001
Baseline CAT [IQI]	11 [5, 19]	14 [7, 21]	<0.001
Baseline SGRQ [IQI]	25 [10, 44]	36 [16, 53]	<0.001
Deaths N (%)	0 (0%)	27 (8.6%)	<0.001

*Kruskal-Wallis test, Chi-square test, or Fisher's exact test comparing participants within each subgroup, as appropriate
Abbreviations: IQI=interquartile interval, FEV_1=forced expiratory volume in 1 second, 6MWD=six-minute walk distance, SGRQ=St. George's Respiratory Questionnaire, CAT=COPD Assessment Test

Supplementary Table 7. P-mtDNA and Disease Progression at 1-Year Follow-Up, Adjusted for Missingness.

	Adjusted for age & smoking status<br>r (CI)*	Adjusted for age, smoking status, and missingness<br>r (CI)*
**FEV₁ % predicted**		
All	-0.01 (-0.09, 0.06)	-0.01 (-0.1, 0.08)
Males	-0.01 (-0.12, 0.09)	-0.03 (-0.13, 0.07)
Male Smokers	-0.08 (-0.27, 0.1)	-0.08 (-1.57, 1.4)
Male Mild/Mod	-0.05 (-0.26, 0.15)	-0.03 (-0.61, 0.54)
Male Severe	0.14 (-0.08, 0.35)	0.09 (-0.28, 0.47)
Females	-0.01 (-0.12, 0.1)	0 (-0.2, 0.2)
Female Smokers	0.04 (-0.16, 0.23)	0.06 (-0.57, 0.68)
Female Mild/Mod	-0.11 (-0.35, 0.13)	-0.07 (-0.97, 0.82)
Female Severe	0.08 (-0.18, 0.34)	-0.03 (-0.74, 0.68)
**6MWD (meters)**		
All	-0.01 (-0.09, 0.07)	-0.03 (-0.19, 0.14)
Males	0.04 (-0.07, 0.15)	0 (-0.16, 0.15)
Male Smokers	-0.01 (-0.18, 0.16)	-0.05 (-0.52, 0.42)
Male Mild/Mod	-0.05 (-0.27, 0.16)	-0.10 (-0.51, 0.32)
Male Severe	0.27 (0.05, 0.48)[a]	0.30 (-0.07, 0.66)
Females	-0.09 (-0.21, 0.04)	-0.21 (-0.67, 0.25)
Female Smokers	0.03 (-0.17, 0.22)	0.03 (-0.25, 0.3)
Female Mild/Mod	0.08 (-0.16, 0.32)	0.12 (-1.6, 1.83)
Female Severe	-0.30 (-0.59, -0.02)[b]	-0.66 (-5.95, 4.63)
**SGRQ**		
All	-0.03 (-0.12, 0.06)	0.01 (-0.11, 0.12)
Males	-0.04 (-0.16, 0.08)	-0.04 (-0.19, 0.11)
Male Smokers	-0.1 (-0.3, 0.1)	-0.07 (-0.74, 0.61)
Male Mild/Mod	0 (-0.2, 0.2)	0.11 (-0.29, 0.5)
Male Severe	-0.16 (-0.41, 0.08)	0.09 (-2.44, 2.61)
Females	-0.01 (-0.14, 0.11)	0.01 (-0.28, 0.31)
Female Smokers	-0.15 (-0.38, 0.08)	-0.15 (-0.84, 0.53)
Female Mild/Mod	0.10 (-0.18, 0.37)	0.20 (-0.43, 0.82)
Female Severe	-0.10 (-0.39, 0.18)	-0.21 (-1.72, 1.29)
**CAT**		
All	-0.07 (-0.15, 0.02)	-0.04 (-0.12, 0.05)
Males	-0.01 (-0.11, 0.1)	0.03 (-0.15, 0.21)
Male Smokers	0.04 (-0.14, 0.21)	0.07 (-1.04, 1.18)
Male Mild/Mod	-0.08 (-0.29, 0.12)	-0.13 (-0.4, 0.13)
Male Severe	-0.05 (-0.27, 0.17)	-0.10 (-0.93, 0.72)
Females	-0.14 (-0.27, -0.01)	-0.14 (-0.44, 0.17)
Female Smokers	-0.30 (-0.52, -0.08)	-0.36 (-0.77, 0.05)

Female Mild/Mod	-0.27 (-0.52, -0.01)	-0.21 (-1.31, 0.89)
Female Severe	0.03 (-0.22, 0.29)	0.05 (-0.54, 0.64)

*Pearson's correlation coefficients with log2 p-mtDNA as among all participants and within each group
[a]Light orange=statistically significant with positive correlation coefficient
[b]Light blue=statistically significant with negative correlation coefficient
Abbreviations: r=correlation coefficient, CI=95% confidence interval, FEV_1=forced expiratory volume in 1 second, 6MWD=six-minute walk distance, SGRQ=St. George's Respiratory Questionnaire, CAT=COPD Assessment Test

Supplementary Table 8. P-mtDNA and Disease Progression at 3-Year Follow-Up, Adjusted for Missingness.

	Adjusted for age & smoking status<br>r (CI)*	Adjusted for age, smoking status, and missingness<br>r (CI)*
**FEV_1 % predicted**		
All	0.04 (-0.06, 0.14)	0.04 (-0.39, 0.46)
Males	0.09 (-0.05, 0.23)	0.03 (-0.43, 0.49)
Females	-0.01 (-0.15, 0.13)	-0.03 (-0.8, 0.73)
**6MWD (meters)**		
All	-0.02 (-0.12, 0.08)	-0.11 (-0.90, 0.69)
Males	0.03 (-0.11, 0.17)	-0.19 (-0.88, 0.50)
Females	-0.07 (-0.23, 0.08)	0.04 (-1.82, 1.90)
**SGRQ**		
All	-0.06 (-0.18, 0.05)	-0.08 (-0.29, 0.14)
Males	-0.07 (-0.22, 0.08)	-0.11 (-0.42, 0.20)
Females	-0.06 (-0.23, 0.10)	-0.02 (-0.48, 0.44)
**CAT**		
All	0 (-0.09, 0.10)	0.05 (-0.07, 0.18)
Males	-0.01 (-0.15, 0.12)	0.07 (-0.15, 0.29)
Females	0.02 (-0.12, 0.17)	0.06 (-0.19, 0.30)

*Pearson's correlation coefficients with log2 p-mtDNA as among all participants and within each group
Abbreviations: r=correlation coefficient, CI=95% confidence interval, FEV_1=forced expiratory volume in 1 second, 6MWD=six-minute walk distance, SGRQ=St. George's Respiratory Questionnaire, CAT=COPD Assessment Test

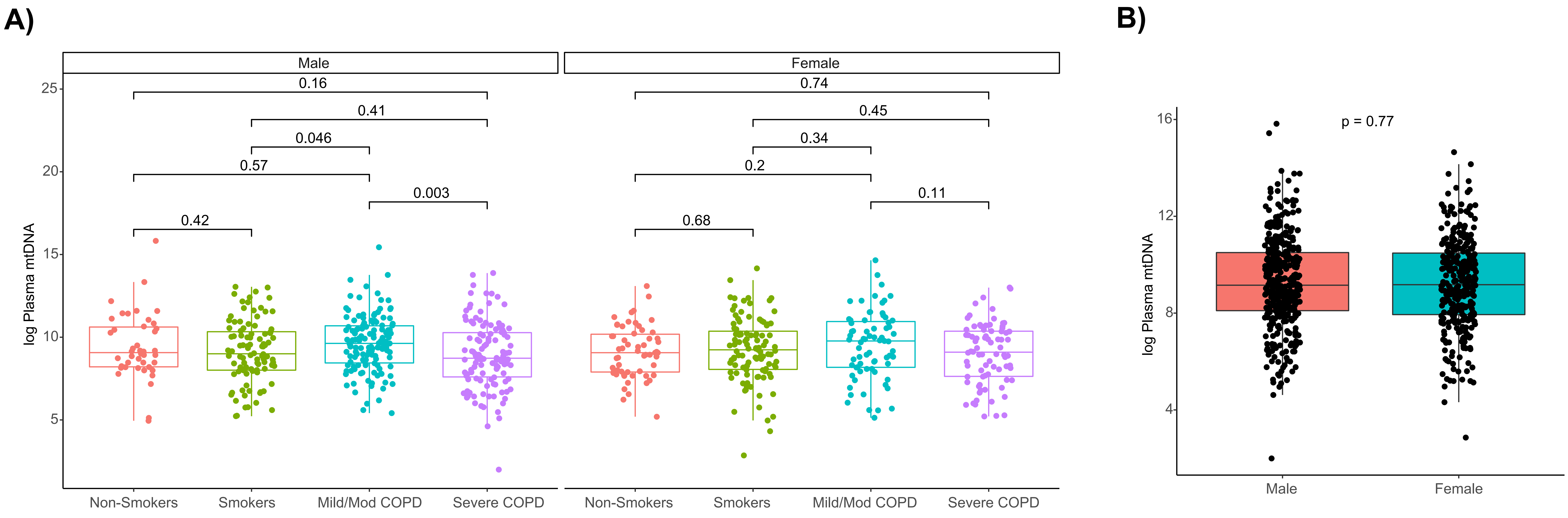

Supplementary Figure 1

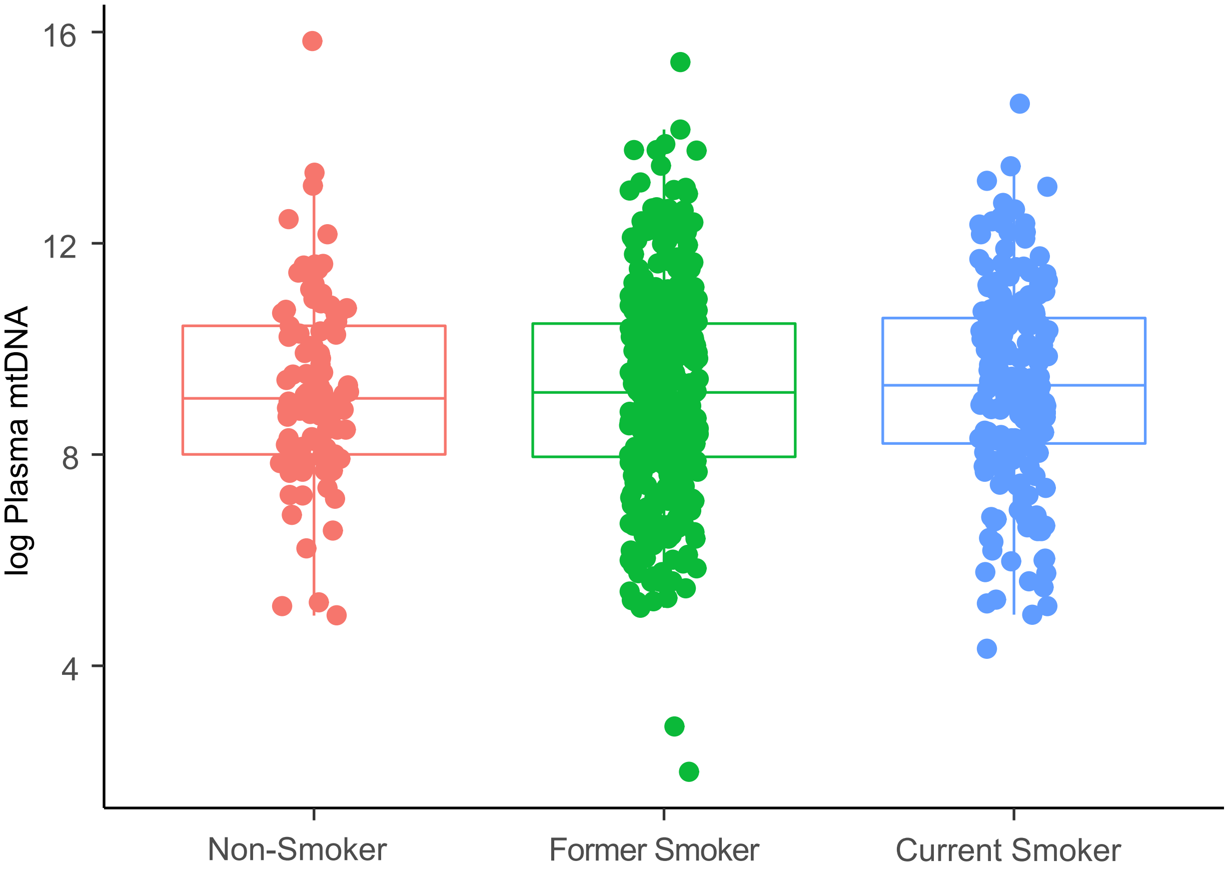

Supplementary Figure 2

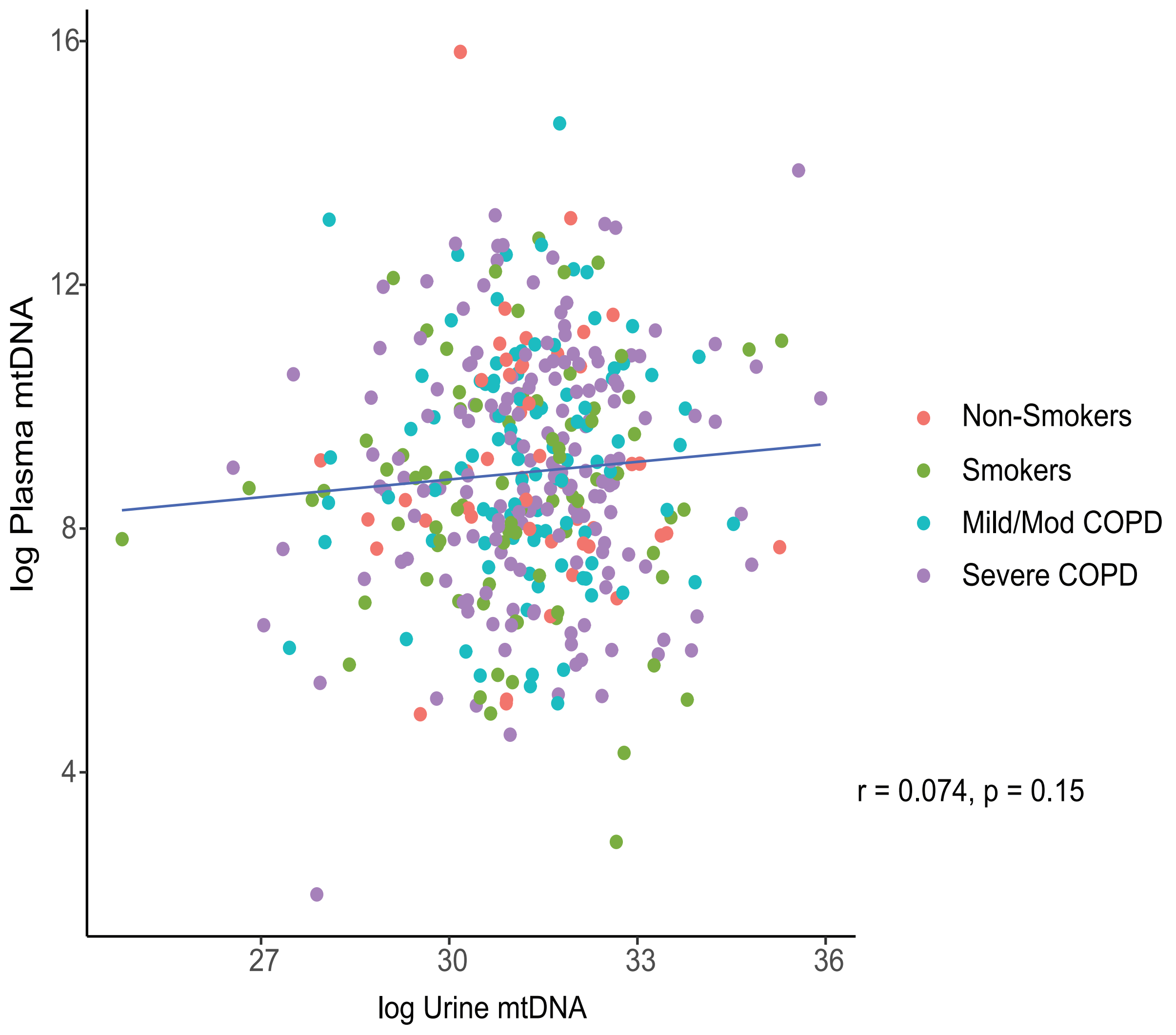

Supplementary Figure 3

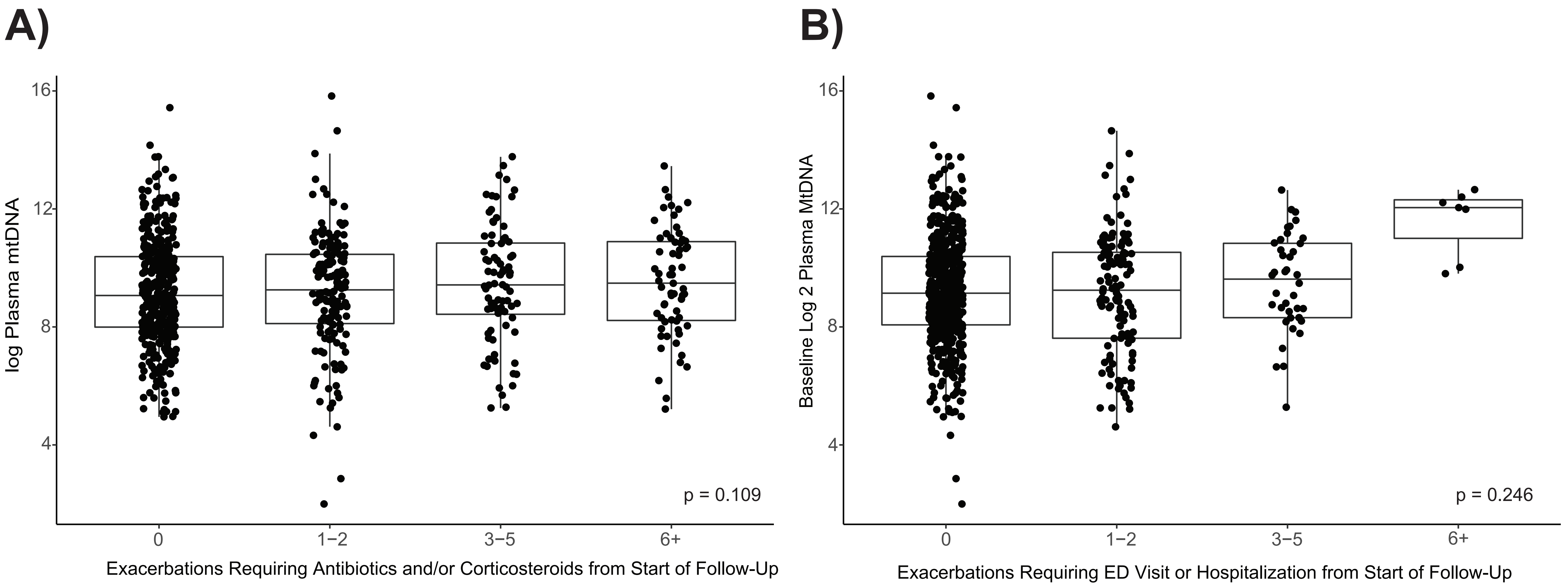

Supplementary Figure 4

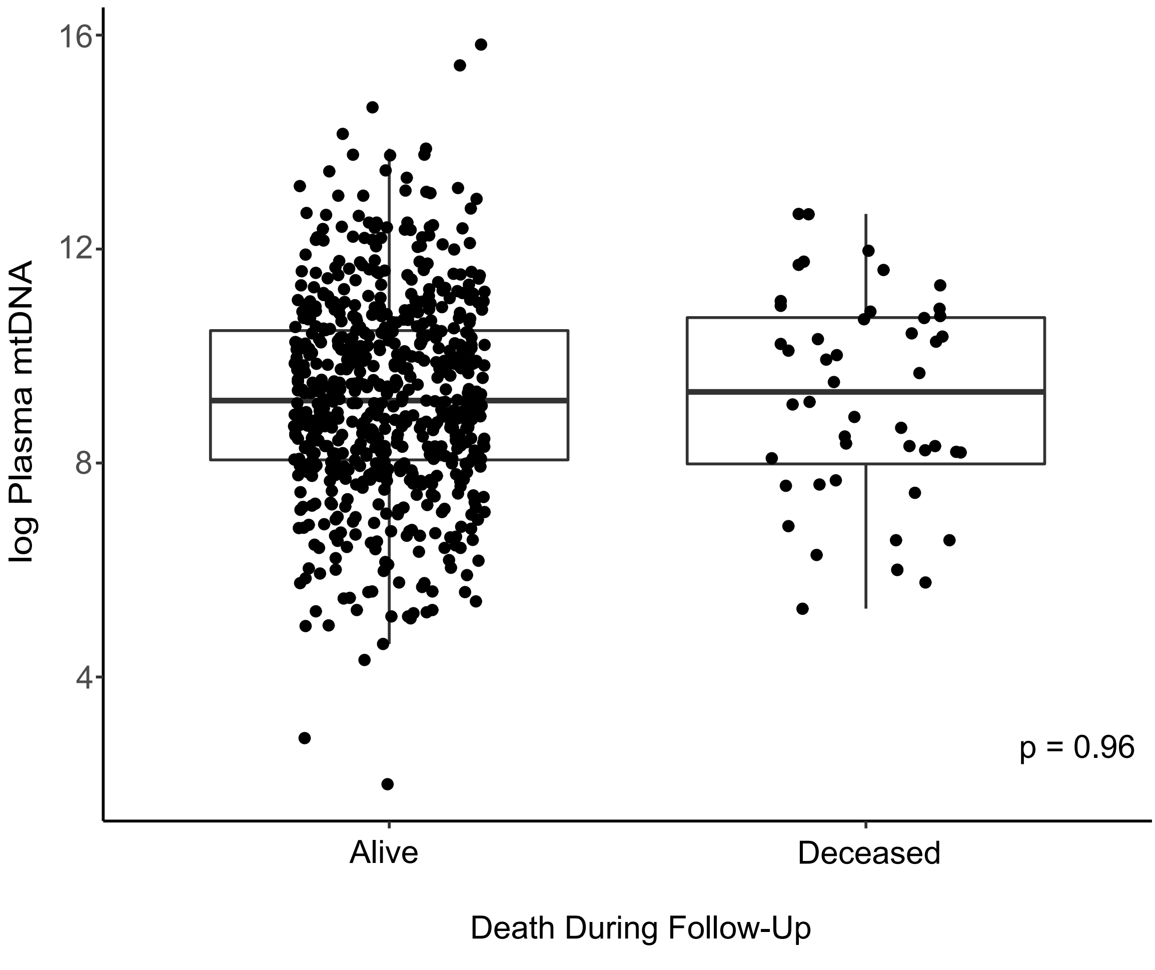

Supplementary Figure 5
